# Parents’ and teachers’ views on sexual health education and screening for sexually transmitted infections among in-school adolescent girls in Kenya: a qualitative study

**DOI:** 10.1186/s12978-017-0360-z

**Published:** 2017-08-14

**Authors:** George Wanje, Linnet Masese, Ethel Avuvika, Anisa Baghazal, Grace Omoni, R. Scott McClelland

**Affiliations:** 1 From the University of Nairobi Institute of Tropical and Infectious Diseases (UNITID), University of Nairobi, P.O Box 91276 - 80103, Mombasa, Kenya; 2 School of Nursing Sciences, University of Nairobi, Kenyatta National Hospital, P.O Box 20804 – 00202, Nairobi, Kenya; 3 Department of Epidemiology, University of Washington, HMC Box 359909, 325 9th Avenue, Seattle, WA 98104–2499 USA; 4 Department of Medicine, University of Washington, HMC Box 359909, 325 9th Avenue, Seattle, WA 98104–2499 USA; 5 Department of Global Health, University of Washington, HMC Box 359909, 325 9th Avenue, Seattle, WA 98104–2499 USA; 6 Ministry of Medical Services, Mombasa County Department of Health, P.O Box 90441 - 80100, Mombasa, Kenya

**Keywords:** Parents, Teachers, Adolescents, Sexual health education, Sexually transmitted infections, Screening, Kenya, Africa

## Abstract

**Background:**

To successfully develop and implement school-based sexual health interventions for adolescent girls, such as screening for *Chlamydia trachomatis, Neisseria gonorrhoeae,* and *Trichomonas vaginalis*, it is important to understand parents’ and teachers’ attitudes towards sexual health education and acceptability of sexually transmitted infection (STI) screening interventions.

**Methods:**

In this qualitative study, we approached parents and teachers from three high schools to participate in in-depth interviews (IDIs) and focus-group discussions (FGDs). Parents and teachers were asked about their general knowledge of STIs and sexual health education. In addition, they were asked whether they would support utilizing outreach to schools to facilitate provision of sexual health education and screening for STIs in adolescent girls. Data were audio-recorded, transcribed, and translated into English. An initial coding matrix was developed and refined throughout the coding process. Transcripts were coded by two researchers and analyzed using the content analysis approach.

**Results:**

We conducted 10 IDIs (5 parents and 5 teachers) and 4 FGDs (2 with parents, 2 with teachers, total of 26 participants). Most parents reported few or no discussions regarding STIs with their adolescent girls. Parents were more comfortable discussing consequences of sexual activity including loss of virginity and the potential for pregnancy. Parents tended to place responsibility for sexual health education with teachers. The teachers, in turn, provided basic sexual and reproductive health education including puberty, abstinence, and overview of STIs. Both parents and teachers found the idea of screening for STIs in adolescent girls to be acceptable, and were comfortable with research staff contacting girls through informational meetings at schools. Parents felt that adolescents’ STI screening results should be shared with their parents.

**Conclusion:**

In this African setting, parents and teachers provide limited sexual health education, with a focus on negative consequences including loss of virginity, pregnancy, and risk for STIs. Nonetheless, both parents and teachers were supportive of STI screening for adolescent girls, beginning with school-based informational meetings for the girls. Research and programs that aim to provide STI screening in this setting must offer treatment and address the issue of whether results will be disclosed to parents.

## Plain english summary

We conducted a qualitative study to understand parents’ and teachers’ attitudes towards sexual health education and acceptability of sexually transmitted infection (STI) screening among adolescent girls in Mombasa, Kenya. We conducted in-depth interviews (IDIs) and focus group discussions (FGDs) with parents and teachers from selected schools. We found that parents rarely discuss sex with their adolescent girls. Teachers provide basic sexual and reproductive health education, following the ‘Life Skills’ educational curriculum from Kenya’s Ministry of Education. Both parents and teachers were supportive of STI screening for adolescent girls in schools. Parents wanted to be informed of test results, which could hinder participation of adolescent girls.

## Background

The global burden of sexually transmitted infections (STIs) remains high, with nearly 1 million curable new infections occurring every day [1]. Low income countries continue to bear the greatest burden of STIs, which may result in negative sexual and reproductive health outcomes including pelvic inflammatory disease, infertility, cervical cancer, fetal and neonatal deaths, and increased HIV risk [2, 3]. In sub-Saharan Africa, the majority of STIs occur in adolescents and young people [4, 5]. The risk associated with younger age has been attributed to early sexual debut, multiple and concurrent sexual relationships, and intergenerational sex.

Many adolescents and young people are sexually active [6], and therefore at risk of contracting STIs. Often, they do not know how to protect themselves from these infections [7]. A cross-sectional survey of STIs among adolescent girls in rural Kenya reported the prevalence of *C. trachomatis*, *N. gonorrhoeae*, and *T. vaginalis* at 2.5%, 0.6% and 2.5% respectively [8]. Data from a cross-sectional study of female adolescents in Uganda estimated the prevalence of *C. trachomatis* at 4.5% [9]. Adolescent girls are more vulnerable to STIs because of predisposing biological and socio-economic factors [10–12]. Sexually transmitted infections may also increase women’s risk of HIV acquisition [13]. These data highlight the importance of STI screening among adolescent girls to keep them healthy [14].

Despite the risky sexual behavior of adolescent girls in Africa [10], social and cultural obstacles impede discussions between adults and adolescents about sexual and reproductive health [15, 16]. Parents are primary socializing agents for their children [17, 18]. However, communication barriers often hinder their ability to provide sexual health knowledge to their adolescent children [19, 20]. A study of parent-adolescent sexual health communication in Ghana highlighted barriers to communication including cultural taboos, adolescent self-image, technology exposure, and poor parental role models [21]. Lack of communication skills and parents’ belief that adolescents are too young to learn about sexual health also contribute to low rates of sexual health discussion [18]. Parents’ reluctance to discuss sexual health could have a significant impact on adolescents’ reproductive health choices [22].

Most studies about adolescents’ sexual health have focused on parents as primary sources of information [19, 21, 23]. Teachers also play an integral socializing role for adolescents attending schools [24], and can have a crucial role in implementing school-based sexual health programs for adolescents [25, 26]. However, teachers face challenges in addressing key topics such as condom use, which may be associated with promiscuity [27]. They may also be uncomfortable discussing subjects like unwanted pregnancies, sexual orientation, masturbation, and contraception [24, 28]. Teachers’ attitudes towards adolescent sexual health education are likely to influence the transfer of sexual reproductive knowledge [27, 29]. In the Kenyan context, the “Life Skills” curriculum is limited, and provides basic sexual health education with a focus on puberty. Some components of reproductive health, like sexuality and reproduction, are covered in other subjects like Biology and Religious Studies [30].

The availability of youth friendly services for reproductive and sexual health is limited in the country. Only 7% of Kenyan health facilities offer access to an adolescent-friendly environment for seeking information and services for sexual health problems including STIs [31]. Screening and treatment for STIs is not free, even in public health facilities. Apart from the paucity of youth friendly health facilities for adolescents, the law also governs the involvement of this understudied vulnerable population in sexual and reproductive interventions. According to Kenya’s National Adolescent Sexual and Reproductive Health Policy 2015, the working definition of an adolescent is a person aged between 10 and 19 years, with the legal age of consent above 18 years [32]. Therefore, for adolescents from 15 to 17 years old to participate in biomedical interventions such as STI screening, parental consent is required in addition to individual assent from the participant. Adolescents from 15 to 17 years old who are married, pregnant, or have children are considered mature minors, and are allowed to provide individual consent (without a parent) according to Kenyan law [33].

To develop effective school-based sexual health interventions which require parental consent, it is imperative to understand both parents’ and teachers’ views on sexual health education, including the challenges they perceive in addressing adolescent sexual health [24, 34]. This qualitative study explored parents’ and teachers’ attitudes towards school-centered sexual health education and promotion of STI screening for adolescent girls (15–17 years) in Mombasa, Kenya. Data from this exploratory qualitative research informed development of a pilot study of a school-based STI screening intervention for *C. trachomatis*, *N. gonorrhoeae*, and *T. vaginalis* in adolescent girls (manuscript submitted).

## Methods

### Study population and procedure

We conducted a qualitative study in Mombasa County, Kenya between June 2013 and April 2014. Participants included parents and teachers from selected educational institutions. We approached several educational institutions and obtained letters of collaboration from three girls high schools. With approval from the institutional leadership, we presented information about the study during meetings of parent-teacher associations. These initial sensitization sessions included information about STIs and an overview of our proposed study. The research team introduced themselves to the parents and teachers. During these meetings, parents and teachers were invited to participate in IDIs and FGDs to understand their attitudes towards sexual health education and screening for STIs in adolescents (ages 15–17 years). The researchers explained that the overarching objective of the study was to evaluate the acceptability of school-based STI screening. Statistics related to STIs among adolescents in Kenya were provided. Since most attendees had not previously participated in research, the informed consent process was explained. A question and answer session followed the presentation. We scheduled follow-up meetings with interested parents and teachers at a convenient time.

We used purposive sampling to recruit parents and teachers for in-depth interviews (IDIs) and focus-group discussions (FGDs), with the aim of obtaining a range of religious, tribal, educational, and socioeconomic backgrounds. The IDIs were intended to elicit individualized stories and perceptions about adolescents sexual health, while the FGDs were employed as an efficient means of collecting data on cultural norms, rather than individual behavior [35]. The questions in the IDIs and FGDs covered the same broad topics on general knowledge of STIs, individual/community attitudes towards STIs and testing, and reproductive health education.

Interested parents and teachers were given consent forms to read and familiarize themselves with the research. All parents and teachers who volunteered to participate in the study were included in the FGDs and IDIs according to their preference. Interviews were conducted at the participants preferred location, and took approximately an hour. Focus group discussions with teachers were conducted at the educational institutions, while FGDs with parents were conducted at our research site, located within Ganjoni Clinic. Socio-demographic information was collected prior to IDIs or FGDs. These activities were conducted by a Kenyan social scientist (GW) fluent in Swahili and English, the official languages in Kenya. We used semi-structured topic guides that allowed for probes and clarification [36]. Topics covered in the IDIs and FGDs included general knowledge about sexual health education, STIs, attitudes towards STIs testing, and willingness of parents to support their adolescent girls in screening for STIs. Participants were asked questions such as “Do you have discussions with your adolescent girl(s)/student(s) about reproductive health issues?”, “What do you know about STIs”, “Would you encourage your adolescent girl to seek testing for STIs”, and “Would you support reproductive health services and STI screening for adolescent girls in school?” All participants were encouraged to use the language they were comfortable either in Swahili or English or a mixture of both. The use of probes allowed the participants to provide more information on any given question and for the researchers to provide clarification where necessary. To ensure that participants understood the study questions, they were invited to ask any questions, and answers were provided by the researcher. Participants were assured information they provided would help in designing a STI screening intervention for adolescent girls. Both parents and teachers were asked what they knew about the benefits of STI testing, and about their knowledge of different screening methods. If participants did not know of any method, the interviewer used probes to seek for further information, such as, “Have you heard of people using genital swabs or urine to test for STIs?” All IDIs and FGDs were audio-recorded, and field notes were taken during the sessions. The interview/FGD session ended with a brief presentation on facts about STIs. Informational handouts about STIs were also distributed. Participants received transportation reimbursement to cover the cost of travel to and from the research activities.

### Data analysis

Audio-recorded IDIs and FGDs were transcribed into Swahili or English, depending on the language used. Swahili text was translated verbatim into English. Software coding using ATLAS.ti (GmbH, version 5.0, Berlin, Germany), and manual coding were performed by two researchers (GW and EA), using an inductive process. The researchers developed an initial code book, guided by the topic guide and research objective. The codebook was refined throughout the coding process. The two researchers separately coded and reviewed all transcripts. Inconsistent results were reviewed by the coders until consensus was reached. Content analysis was used to develop and group themes emerging from the IDIs and FGDs [37, 38].

We received ethical approval from the Kenyatta National Hospital-University of Nairobi Ethics and Research Committee (KNH-UON ERC) and the Human Subjects Research Committee (HSRC) at University of Washington. Written informed consent was obtained from all participants.

## Results

### Participant characteristics

We conducted a total of 10 IDIs (5 with parents, and 5 with teachers), and 4 FGDs (2 with parents and 2 with teachers), with a total of 26 participants from the three educational institutions. Detailed participant characteristics are presented in Table 1. The majority of parents and teachers were female and married. The results are presented by main themes, supported by quotations voiced by parents and teachers.

**Table 1:** Baseline characteristics of in-depth interview and focus group discussion participants

Characteristic	Median (IQR) or Number (percent)	
In-depth Interview Participants	Parents (*n* = 5)	Teachers (*n* = 5)
Age (years)	40 (38–53)	41 (40–42)
Female	4 (80%)	5 (100%)
Married	4 (80%)	4 (80%)
Education Level		
Primary	3 (60%)	0 (0%)
Secondary	2 (40%)	0 (0%)
College	0 (0%)	0 (0%)
Postgraduate	0 (0%)	5 (100%)
Religion		
Christian	2 (40%)	3 (60%)
Muslim	3 (60%)	2 (40%)
Number of children	3 (2–3)	3 (2–3)
Source of Income		
Unemployed	2 (40%)	0 (0%)
Employed	0 (0%)	5 (100%)
Self-employed	3 (60%)	0 (0%)
Focus Group Discussion Participants	Parents (*n* = 9)	Teachers (*n* = 17)
Age (years)	42 (39–45)	30 (27–40)
Female	6 (67%)	9 (53%)
Married	7 (78%)	12 (71%)
Education Level		
Primary	0 (0%)	0 (0%)
Secondary	5 (56%)	0 (0%)
College	2 (22%)	8 (47%)
Postgraduate	2 (22%)	9 (53%)
Religion		
Christian	6 (67%)	14 (82%)
Muslim	3 (33%)	3 (18%)
Number of children	3 (1–4)	1 (0–2)
Source of Income		
Unemployed	0 (0%)	0 (0%)
Employed	5 (56%)	17 (100%)
Self-employed	4 (44%)	0 (0%)

### Main themes

Four main themes emerged from the data. These were categorized as lack of sexual health discourse, parents’ and teachers’ knowledge about STIs, STI screening acceptability, and disclosure of STI screening results.

### Lack of sexual health discourse

Most parents acknowledged that they rarely discussed sexual health with their adolescent girls. When discussion of sexual health occurred, they did not talk about STIs. Many parents felt that their adolescent girls should not engage in any sexual activity prior to marriage. Accordingly, discussions about sex most often addressed loss of virginity and the risk of pregnancy.

To be honest, there is not a lot of talk about sexually transmitted diseases. First of all, we don’t want them to engage in sex.

(38 year old female parent, IDI)

Personally when we talk to our children, we tell them not to engage in the act [sex] at all, and to wait for the right time, because if they are tricked and impregnated, many [men] will not take responsibility for the pregnancy since their main interest was sex and not the person. We tell them, “If you find someone who likes you and you like them, let him come home and ask for your hand in marriage. Make it formal”. So we insist that act [sex] is only for married people.

(60 year old male parent, IDI)

Most female parents reported being more engaged with their adolescent girls’ sexual health when the topic arises as opposed to the male parents. One female parent added that mother-daughter relationships determines the depth of discourse between the two. Some fathers noted that the African culture and even religion prevented them from being close with their adolescent girls.

According to us mothers, we feel it is not easy for fathers to discuss girls “personal issues”. It is the duty of the mother, unless there is a situation where the girl has become pregnant then he becomes angry, but otherwise it is not easy for fathers to talk freely just like that to girls.

(42 year old female parent, FGD)

In African setting and culture, it a taboo to talk to your child about sex, especially my daughter. I normally buy educative books for her, but discussions are directed to the mother.

(54 year old female parent, FGD)

I think it’s mainly because of religion, because you will find that personally when my daughter is on her periods [menstruating] you will find her mother telling me to buy her things [pads] or mostly when you go to the supermarket you will see the mother getting it and putting it in the basket (*laughs*). You will see the communication is sort of remote and not direct and that is how you will know [she has started menstruating].

(60 year old male parent, IDI)

Teachers believed that parents did not talk with adolescent girls about sexual health, or avoided more sensitive topics like STIs. The teachers seemed aware that parents focused primarily on the risk of pregnancy, a more socially acceptable discussion topic.

You know, for some parents, it is a taboo to discuss sexual issues with their children*.*

(40 year old female teacher, IDI)

The parents still fear to talk about the issues concerning sex with their daughters. So they tell them you are not supposed to do this, and this, and this, but they don’t give a reason as to why. The [parents] tell their girls, “You are going to get pregnant.” But after that, they are not talking about the other issues and complications concerning STIs.

(42 year old male teacher, FGD)

Most teachers felt that parents shift responsibility for discussing reproductive health issues to the teachers. In response, they provide basic information on sexual health according to the Kenyan educational curriculum.

Even our mothers did not have time to tell us about sexuality, simply because they knew we were supposed to learn about them either in school or in “madrasa”. So the issue of parents talking to children about sex does not happen. The parents will say, “Don’t play around with boys or else you will get pregnant.” But they will not tell their girls about other aspects of sexuality.

(40 year old female teacher, IDI)

‘Life skills’ is part of the curriculum that was introduced by the Ministry of Education in schools, so as to help students in learning. It is like social studies that were before. So they learn the normal skills in life, self-esteem, and a little of reproductive health. I don’t think there is much of reproductive health content in that.

*(28 year old male teacher, FGD*)

### Parents’ and teachers’ knowledge about STIs

Most of the parents believed that they had inadequate STI knowledge, including lack of knowledge about different types of infections and how they are transmitted. Common STIs that parents were able to remember included syphilis, gonorrhea and HIV. Parents were asked to remember the STIs they knew, and probed further to establish their accuracy in naming STIs and description of signs and symptoms. The most frequent themes during the IDIs and FGDs related to limited knowledge on the subject of STIs. One participant noted that adolescent girls can acquire STIs through low standards of hygiene and not necessarily sexually. Some participants held misconceptions about STIs being transmitted by dirty water or sharing toilets.

What I know is there are several types of STIs. If you sit on a toilet where someone who is infected has used, especially the sitting toilets you get these STIs.

(44 year old male parent, FGD)

I have not heard a lot about STIs, but in the past we used to hear a lot about mmh, I don’t know. Bilharzia [schistosomiasis], that’s the disease, yeah. I think something like that. If you are with a man who has it, they will infect you and you will urinate blood. Something like that, that’s the one STI I remember.

(45 year old male parent, FGD)

From what I know, I believe gonorrhea is transmitted through direct intercourse, and unprotected sex. Syphilis can also be gotten through unprotected sex as well as the latrines. Maybe one of you has the disease and their urine got on the latrine seat. The disease is not something that dies off quickly, so when you go and sit on the toilet seat, you also get infected.

(38 year old female parent, IDI)

Most teachers especially those who taught Biology seemed to be knowledgeable about STIs, including recognizing that some infections are asymptomatic. However, some teachers had misconceptions on how STIs are transmitted. One teacher noted that sharing of toilet seats and clothing would result in transmission of STIs. Teachers were also aware of the negative health consequences of untreated STIs. Some of the teachers were aware that STIs can be asymptomatic. However, most of them felt that they needed more information on sexual health, including STIs, to enable them to adequately address the questions posed by their students.

Well right now because I have learnt a bit of the STI’s, I know men do get the symptoms early, they get to know of the infection earlier than the women. For the women it takes some time. So for those men who are bright enough they will even seek treatment earlier before even their wives have gone to be treated mmhh.

(41 year old female teacher, IDI)

They can either be sexually transmitted, or you can either get them through things like sharing toilet seats or in some small percentages sharing things like swimming costumes. Yeah.

(27 year old female teacher, FGD)

To tell you the truth, I know of syphilis, gonorrhea and HIV. I have forgotten the others apart from those. These three are the ones that are usually talked about more so even if there are others they are not as strong to stick in your mind.

(40 year old female teacher, IDI)

Even in the class while teaching, we biology teachers sometimes are asked questions until you wonder, and because you are not a doctor you push the question forward to the next lesson so that you go and research.

(46 year old male teacher, FGD)

### STI screening acceptability

Screening for STIs in adolescent girls was considered to be acceptable by all of the parents and teachers. Many parents suspected that their adolescent girls were engaging in sexual relations, especially a common practice called “brushing” (non-penetrative sex), which is meant to preserve virginity. Parents reported that screening would inform them if their children had STIs. As parents, they felt that it was their role to facilitate treatment, if needed. All of the parents in our IDIs and FGDs reported that they would be willing to sign the consent forms allowing their adolescent girls to participate in STI screening.

I can tell you most of them [adolescent girls] are having sex, because you will find many of them know about contraceptives. Why would you use contraceptives if you did not have sex? So that is a sign showing that many of them have started doing it [having sex] very early.

(40 year old female parent, IDI)

I think they should be tested, because they may have a boyfriend and you do not know [as a parent] … However, there are these stories of ‘brushing’ on top. She may get a boyfriend and they go together and do it saying, “Ah there is nothing wrong as this is just ‘brushing.”

(36 year old female parent, IDI)

I will sign for her the consent because to say the truth bringing up a child I can say it is not easy, it is very hard. Being open is very important.

(38 year old female parent, IDI)

Both parents and teachers acknowledged there could be potential stigma for adolescent girls who screen for STIs. Most of the participants felt that STIs are associated with immorality. Adolescent girls who undergo STI screening might be labelled as promiscuous. Participants also mentioned that the screening intervention should not be done in schools, to minimize stigmatization of the girls by their teachers and peers.

“It is important for girls to be tested through schools but other parents might think the reason the daughter is volunteering is because she is doubting herself.”

(59 year old female parent, FGD)

I think there is stigma on their sexuality and morality, because somebody is being tested for STIs. Their morality is very questionable.

(27 year old male teacher, FGD)

I think aah if they are tested in schools it can bring controversy. It can bring some kind of pandemonium (*laughter*). Hospital set up is better because here [in schools] they might also suffer victimization from their colleagues who are not being tested. So even if they volunteer, those who do not volunteer will start giving them nicknames.

(25 year old male teacher, FGD)

Besides accepting the STI screening, the parents’ narratives emphasize the need to have pre-intervention information sessions with adolescent girls to address misconceptions regarding STIs.

For many, the information on STI is lacking. It is an important matter to give them the information and alerting them on what is going on, and they will respond to the screening.

(53 year old female parent, IDI)

[Other students and teachers] will assume she’s promiscuous. They will question; “Why are you screening?”, “Are you promiscuous?”, “Do you exchange men?”, “Are you loose?” That is how it will be if the girls are not informed about the research properly.

(44 year old female parent, FGD)

Teachers did not have any objection to the development of a STI screening intervention for adolescent girls in their schools, provided that parental consent was obtained. Additionally, teachers stressed that the screening intervention should be voluntary, allowing adolescent girls to make informed decisions on whether they would like to get tested for STIs.

It is fine to test them as long as you have consent from the parents. If you do not have consent, you cannot test them.

(27 year old female teacher, FGD)

I think if they are to be screened, it should be voluntary. The girls should be informed and asked if they are ready.

(46 year old male teacher, FGD)

### Disclosure of STI screening results

Parents were willing to allow their adolescent girls to participate in the STI screening intervention on condition that the results would be disclosed to the parents. They wanted to be informed of either positive or negative results, and to take an active role in ensuring treatment if STIs were detected.

I want to know [the results], so I can know how to handle her if she has an infection. There was a day she had a pimple in her private parts and she told me, “mum come and see I have a pimple.” I went and checked her and told her, “It is just a boil.” If she has been tested and I don’t know the results, then there is no point in her getting tested. I should just have left her as she is.

(36 year old female parent, FGD)

If I signed for her [the consent], it means I want to know the results. If I wouldn’t have signed, it means I wouldn’t want to know. Why would I sign her consent form and not want to know the outcome?

(38 year old female parent, IDI)

Some teachers felt it was important to know the aggregated, but not individual, results from STI screening in the schools. They believed that this information would enable them to provide more accurate sexual health information to their students.

I think it is better if the results are kept confidential and not shared with teachers. Because, like she said, stigmatization is very high, hence a high chance of stigmatizing the student. That would bring them down and they might not even learn. Some might even commit suicide, so knowing their test results might have dire consequences.

(42 year old female teacher, FGD)

We may ask for statistics from our school like, eeh, “Can you give us an estimate or a percentage of who are like this or who are like this” … And it might be good for guidance and counseling, or it might be taken on the negative side by the school community.

(25 year old male teacher, FGD)

## Discussion

In this qualitative study, we found that parents had limited knowledge of STIs and rarely discussed them with their adolescent girls. Nonetheless, they expressed willingness to have their adolescent girls participate in STI screening, and wanted to be informed of the screening results. Teachers were somewhat more knowledgeable about STIs. They were also supportive of STI screening for adolescent girls, including recruitment from schools, provided that parental consent was obtained. We did not find substantial differences in views about adolescents’ sexual health and acceptability of STI screening by parents’ or teachers’ gender, religion, education level, or socioeconomic background. There were also no differences in participants’ expectations concerning adolescent girls’ sexual health from the IDIs and FGDs.

The results from this study are consistent with findings from a systematic review of studies conducted in sub-Saharan Africa [17]. The review found that parental discussions regarding sex tend to be general, and characterized by warnings, rather than direct and open discussion. Most parents in our study gave warnings about preserving virginity and avoiding pregnancy. They believed that their daughters should not engage in sex before marriage, but also seemed to be aware that many adolescent girls are sexually active. Our results highlight the need for improving parenting skills through development of interventions geared towards parent-adolescent communication around sexual health in Africa. Such interventions could form a foundation for stronger sexual health programming for adolescents [39]. Parents felt that the responsibility for providing sexual health education lay primarily with teachers. This may be because of the taboo nature of discussing sex [17, 18], which negatively impacts sexual health communication between parents and adolescents. Empowering parents and teachers to provide sexual health information to adolescent girls, including screening and treatment for STIs, could have a positive impact to adolescents’ health. This benefit would, in turn, complement the US President’s Emergency Plan for AIDS Relief (PEPFAR) goal in implementing the Determined, Resilient, Empowered, AIDS-free, Mentored, and Safe women program (DREAMS) program, which aims to reduce new HIV infections among adolescent girls and young women in 10 sub-Saharan African countries.

The majority of parents did not know the different types of STIs, their symptoms, or how they are transmitted. Their reluctance to discuss STIs with their adolescent girls may relate, at least in part, to their own limited STI knowledge. All misconceptions about mode of transmission for STIs, the different types of STIs, and any questions from participants were fully addressed at the end of the interviews and FGDs. A study conducted in the United States showed that greater knowledge on human papillomavirus (HPV) of mothers of adolescent girls resulted in more conversations on the vaccine and sexual health with their daughters [40]. In contrast to parents, teachers were more knowledgeable about STIs. Nonetheless, they reported the desire for additional information to better equip them for discussions with their students. A review and revision of the existing Kenyan Life Skills Education curriculum to holistically address sexual health education could help to bridge the knowledge gap that exists among teachers, allowing them to provide additional important sexual health information to adolescents. These results from Mombasa, Kenya highlights the need for interventions aimed at increasing STI education for parents and teachers, as detailed in earlier work from other locations [41–43].

While parents were willing to support adolescent girls’ participation in STI screening, all parents in our study stated that they would want to know the test results. The main reason was to ensure that girls were treated if STIs were identified. In a previous study that we conducted among adolescents from the same schools, we found that disclosure of adolescents’ STI screening results to their parents is a controversial topic [44]. Addressing the potentially conflicting desires of adolescents and their parents is an important challenge that must be overcome if STI screening is to be provided to minors who require parental consent to participate [45, 46].

Parental consent is not required for adolescents from the age of 15 years seeking HIV testing on their own in Kenya [47]. The recent change in HIV Testing Guidelines has led to approximately 50% of Kenyan adolescents 15–19 years old knowing their HIV status [33]. Nevertheless, some health care providers still require parental consent, and this can be a barrier to HIV testing. Adolescents in the country can also access contraception services without parental consent [32]. However, guidelines differ when it comes to adolescents’ participation in health research, rather than participation in programs. Adolescents under the age of 18 years cannot legally participate in research without parental/guardian consent unless they are emancipated minors [33].

Adolescent girls’ participation in STI screening could be adversely impacted if they felt their test results would be shared with parents who were unaware that they were sexually active. In preparation for a preliminary STI screening exercise in schools in Mombasa, we sought guidance from the KNH-UON ERC on this issue. The committee recommended that written parental consent should clarify that the research team would not share results with parents. Instead, the adolescent girl would decide whether to disclose her results. Because parents were particularly concerned about the need to treat any STIs detected, screening interventions must offer treatment, as it would be unethical to test and not offer treatment. In our study, adolescent girls were offered free STI screening. In the event of a positive test result for any STI, treatment was provided at no charge according to Kenyan National Guidelines for treatment of STIs. We also offered free counseling and treatment to sexual partners.

Few studies have focused on STI screening acceptability in Africa. The qualitative data collected in this study provided us with preliminary data that were well suited to our research objective, which was to pilot test a school-centered STI screening intervention for adolescent girls. In a follow-up study, these rich data informed the development of a successful school-based STI screening intervention. Adolescent girls’ urine samples were tested for the presence of *C. trachomatis*, *N. gonorrhoeae* and *T. vaginalis* by transcription mediated amplification using the Hologic Aptima Detection System (Hologic, San Diego, CA, USA) (manuscript submitted). The use of 20–30 milliliter (ml) first-catch urine specimen to test all the three STIs led to wider acceptance and success of the intervention. Inclusion of both parents and teachers afforded us a richer understanding of sexual health in this setting.

This study also had limitations. First, we recruited participants from three high schools in urban Mombasa, so our findings may not be generalizable to rural settings. Second, parents and teachers who were willing to participate in the study might be different from those who did not participate. Third, our study did not address parents’ and teachers’ views on adolescent HIV testing. Importantly, the current HTS guidelines in Kenya allow adolescents to seek HIV testing without parental consent, but this guidance does not apply to other STIs. Fourth, we focused on girls because they typically initiate sex earlier [5], and have greater morbidity associated with STIs [48, 49], compared to boys. Nonetheless, we acknowledge that some boys may be at risk for STIs, and that our data may not reflect parents’ and teachers’ views on STI screening in male adolescents. Despite the relatively small number of interviews and FGDs in this study, we did not find new themes across the conducted FGDs and IDIs. We also triangulated our data with 10 IDIs to enrich our understanding of parents’ and teachers’ views towards adolescent girls’ sexual health. Data from this study were important, and suitable for planning an STI screening pilot study. We also felt that these data would be of interest to other researchers and program planners with an interest in screening for STIs or addressing adolescent sexual health in similar populations.

Despite the limitations noted, we feel that the study provides valuable insight about parents’ and teachers’ attitudes towards adolescent girls’ sexual health and STI screening. In general, we feel that STIs and their morbidity are an important consideration for adolescent girls in Africa. However, we recognize that additional research on the prevalence STIs in this population, as well as the costs and budget impact of different screening strategies are needed to make informed recommendations about providing services on a broader scale. Our study results highlighting parents’ and teachers’ views on the acceptability of STI screening suggest that expanded interventions are possible in this community. Additional work is needed to explore the acceptability of potentially more sensitive topics including HIV testing and provision of contraception.

## Conclusion

Given the high incidence of STIs risk among African adolescent girls [49, 50], it is encouraging that both parents and teachers were supportive of school-centered STI screening for adolescent girls. Addressing the issue of disclosure versus non-disclosure of STI screening results to parents is likely to be a cross-cutting issue for research and programs addressing STIs in adolescents throughout Africa. The optimal approach will depend on the social and legal context of different settings.

## Abbreviations

FGD: Focus Group Discussion
HSRC: Human Subjects Research Committee
IDI: In-depth Interview
KNH-UON ERC: Kenyatta National Hospital-University of Nairobi Ethics and Research Committee
STI: Sexually Transmitted Infection

## References

[1] Newman L, Rowley J, Vander Hoorn S, Wijesooriya NS, Unemo M, Low N, Stevens G, Gottlieb S, Kiarie J, Temmerman M (2015). Global estimates of the prevalence and incidence of four curable sexually transmitted infections in 2012 based on systematic review and global reporting. PLoS One.

[2] Ebrahim S, Mndende XK, Kharsany AB, Mbulawa ZZ, Naranbhai V, Frohlich J, Werner L, Samsunder N, Karim QA, Williamson AL (2016). High burden of human Papillomavirus (HPV) infection among young women in KwaZulu-Natal**,** South Africa. PLoS One.

[3] Hegdahl HK, Fylkesnes KM, Sandoy IF (2016). Sex differences in HIV prevalence persist over time: evidence from 18 countries in sub-Saharan Africa. PLoS One.

[4] Rassjo EB, Kambugu F, Tumwesigye MN, Tenywa T, Darj E (2006). Prevalence of sexually transmitted infections among adolescents in Kampala, Uganda, and theoretical models for improving syndromic management. J Adolesc Health.

[5] Zaba B, Pisani E, Slaymaker E, Boerma JT (2004). Age at first sex: understanding recent trends in African demographic surveys. Sex Transm Infect.

[6] Marston M, Beguy D, Kabiru C, Cleland J (2013). Predictors of sexual debut among young adolescents in Nairobi’s informal settlements. Int Perspect Sex Reprod Health.

[7] Tenkorang EY, Maticka-Tyndale E (2014). Individual- and community-level influences on the timing of sexual debut among youth in Nyanza, Kenya. Int Perspect Sex Reprod Health.

[8] Kerubo E, Laserson KF, Otecko N, Odhiambo C, Mason L, Nyothach E, Oruko KO, Bauman A, Vulule J, Zeh C (2016). Prevalence of reproductive tract infections and the predictive value of girls’ symptom-based reporting: findings from a cross-sectional survey in rural western Kenya. Sex Transm Infect.

[9] Rassjo EB, Mirembe FM, Darj E (2006). Vulnerability and risk factors for sexually transmitted infections and HIV among adolescents in Kampala, Uganda. AIDS Care.

[10] Darj E, Mirembe FM, Rassjo EB (2010). STI-prevalence and differences in social background and sexual behavior among urban and rural young women in Uganda. Sex Reprod Healthc.

[11] Leclerc-Madlala S (2008). Age-disparate and intergenerational sex in southern Africa: the dynamics of hypervulnerability. AIDS.

[12] Longfield K, Glick A, Waithaka M, Berman J (2004). Relationships between older men and younger women: implications for STIs/HIV in Kenya. Stud Fam Plan.

[13] McClelland RS, Sangare L, Hassan WM, Lavreys L, Mandaliya K, Kiarie J, Ndinya-Achola J, Jaoko W, Baeten JM (2007). Infection with Trichomonas vaginalis increases the risk of HIV-1 acquisition. J Infect Dis.

[14] Cohen MS, Eron JJ (2011). Aciclovir treatment for human immunodeficiency virus-1: is the "juice worth the squeeze?". Sex Transm Dis.

[15] Rassjo EB, Darj E (2002). "safe sex advice is good - but so difficult to follow". Views and experiences of the youth in a health centre in Kampala. From Kiswa youth clinic, Kampala, Uganda. Afr Health Sci.

[16] Cherie A, Berhane Y: Oral and anal sex practices among high school youth in Addis Ababa**,** Ethiopia BMC Public Health 2012, 12:5. 10.1186/1471-2458-12-5 PMC3265418 22216887

[17] Bastien S, Kajula LJ, Muhwezi WW (2011). A review of studies of parent-child communication about sexuality and HIV/AIDS in sub-Saharan Africa. Reprod Health.

[18] Ayalew M, Mengistie B, Semahegn A (2014). Adolescent-parent communication on sexual and reproductive health issues among high school students in Dire Dawa, eastern Ethiopia: a cross sectional study. Reprod Health.

[19] Berg K, Sun CJ, Babalola S (2012). Predictors of parent-child communication among a nationally representative sample in Nigeria. SAHARA J.

[20] Johnson SD, Williams SL (2015). Solution-focused strategies for effective sexual health communication among African American parents and their adolescents. Health Soc Work.

[21] Asampong E, Osafo J, Bingenheimer JB, Ahiadeke C (2013). Adolescents and parents’ perceptions of best time for sex and sexual communications from two communities in the eastern and Volta regions of Ghana: implications for HIV and AIDS education. BMC Int Health Hum Rights.

[22] Lebese RT, Davhana-Maselesele M, Obi CL (2010). Sexual health dialogue between parents and teenagers: an imperative in the HIV/AIDS era. Curationis.

[23] Iliyasu Z, Aliyu MH, Abubakar IS, Galadanci HS (2012). Sexual and reproductive health communication between mothers and their adolescent daughters in northern Nigeria. Health Care Women Int.

[24] Aransiola JO, Asa S, Obinjuwa P, Olarewaju O, Ojo OO, Fatusi AO (2013). Teachers’ perspectives on sexual and reproductive health interventions for in-school adolescents in Nigeria. Afr J Reprod Health.

[25] Schutte L, van den Borne M, Kok G, Meijer S, Mevissen FE (2016). Innovatively supporting Teachers’ implementation of school-based sex education: developing a web-based coaching intervention from problem to solution. J Med Internet Res.

[26] Akbari Kamrani M, Yahya SS (2016). Bringing X, Y, Z generations together to facilitate school-based sexual and reproductive health education. Glob J Health Sci.

[27] Ahmed N, Flisher AJ, Mathews C, Jansen S, Mukoma W, Schaalma H (2006). Process evaluation of the teacher training for an AIDS prevention programme. Health Educ Res.

[28] Mkumbo KA (2012). Teachers’ attitudes towards and comfort about teaching school-based sexuality education in urban and rural Tanzania. Glob J Health Sci.

[29] DePalma R, Francis D (2014). South African life orientation teachers: (not) teaching about sexuality diversity. J Homosex.

[30] Kenya Ministry of Education USAfID, FHI, The United Nations Educational, Scientific and Cultural Organization (UNESCO) (2010). Life skills education in Kenya: a comparative analysis and stakeholder perspective.

[31] National Coordinating Agency for Population and Development (NCAPD) [Kenya] Mo, Medical Services (MOMS) [Kenya] MoPHaSMK, Kenya National Bureau of Statistics (KNBS) [Kenya] IM: Kenya Service Provision Assessment Survey 2010. In. Edited by and NCAfP, Development MoMS, Ministry of Public Health and Sanitation, Kenya, National Bureau of Statistics aIM; 2011.

[32] Ministry of Health K (2015). National Adolescent Sexual and reproductive health policy.

[33] National AIDS and STI Control Programme (NASCOP) and Kenya Medical Research Institute (KEMRI). Guidelines for conducting adolescent HIV sexual and reproductive health research in Kenya. Ministry of Health Government of Kenya; 2015.

[34] Eisenberg ME, Madsen N, Oliphant JA, Sieving RE (2013). Barriers to providing the sexuality education that teachers believe students need. J Sch Health.

[35] Halcomb EJ, Gholizadeh L, DiGiacomo M, Phillips J, Davidson PM (2007). Literature review: considerations in undertaking focus group research with culturally and linguistically diverse groups. J Clin Nurs.

[36] Strauss AJC (2008). Basics of qualitative research: techniques and procedures for developing grounded theory.

[37] Pope C, Ziebland S, Mays N (2000). Qualitative research in health care. Analysing qualitative data. BMJ.

[38] Sandelowski M, Barroso J (2003). Classifying the findings in qualitative studies. Qual Health Res.

[39] Knerr W, Gardner F, Cluver L: Improving positive parenting skills and reducing harsh and abusive parenting in low- and middle-income countries: a systematic review. Prev Science 2013, 14(4):352-363. 10.1007/s11121-012-0314-1 23315023

[40] McRee AL, Reiter PL, Gottlieb SL, Brewer NT (2011). Mother-daughter communication about HPV vaccine. J Adolesc Health.

[41] Okereke CI (2010). Unmet reproductive health needs and health-seeking behaviour of adolescents in Owerri, Nigeria. Afr J Reprod Health.

[42] Sutton MY, Lasswell SM, Lanier Y, Miller KS (2014). Impact of parent-child communication interventions on sex behaviors and cognitive outcomes for black/African-American and Hispanic/Latino youth: a systematic review, 1988-2012. J Adolesc Health.

[43] Taylor M, Joshi A (2012). Surveys assessing STI related health information needs of adolescent population. Technol Health Care.

[44] Avuvika E, Masese LN, Wanje G, Wanyonyi J, Nyaribo B, Omoni G, Baghazal A, McClelland RS (2017). Barriers and facilitators of screening for sexually transmitted infections in adolescent girls and young women in Mombasa, Kenya: A Qualitative Study. PLoS One.

[45] Ott MA, Rosenberger JG, Fortenberry JD (2010). Parental permission and perceived research benefits in adolescent STI research. J Empir Res Hum Res Ethics.

[46] DiClemente RJ, Ruiz MS, Sales JM (2010). Barriers to adolescents’ participation in HIV biomedical prevention research. J Acquir Immune Defic Syndr.

[47] Ministry of Health NAaSCPN (2015). The Kenya HIV testing services guidelines.

[48] Crucitti T, Jespers V, Mulenga C, Khondowe S, Vandepitte J, Buve A (2010). Trichomonas vaginalis is highly prevalent in adolescent girls, pregnant women, and commercial sex workers in Ndola**,** Zambia. Sex Transm Dis.

[49] Hokororo A, Kihunrwa A, Hoekstra P, Kalluvya SE, Changalucha JM, Fitzgerald DW, Downs JA (2015). High prevalence of sexually transmitted infections in pregnant adolescent girls in Tanzania: a multi-community cross-sectional study. Sex Transm Infect.

[50] Joseph Davey DL, Shull HI, Billings JD, Wang D, Adachi K, Klausner JD (2016). Prevalence of curable sexually transmitted infections in pregnant women in low- and middle-income countries from 2010 to 2015: a systematic review. Sex Transm Dis.

